# Tunable Double-Network GelMA/Alginate Hydrogels for Platelet Lysate-Derived Protein Delivery

**DOI:** 10.3390/bioengineering10091044

**Published:** 2023-09-05

**Authors:** Andrea Marfoglia, Fahd Tibourtine, Ludovic Pilloux, Sophie Cazalbou

**Affiliations:** 1 CIRIMAT, Université Toulouse 3 Paul Sabatier, Toulouse INP, CNRS, Université de Toulouse, 31062 Toulouse, France; andrea.marfoglia@univ-tlse3.fr (A.M.);; 2 Laboratoire de Génie Chimique, Université Toulouse 3 Paul Sabatier, Toulouse INP, CNRS, Université de Toulouse, 31062 Toulouse, France; ludovic.pilloux@univ-tlse3.fr

**Keywords:** tissue engineering, regenerative medicine, hydrogels, GelMA, alginate, platelet lysate

## Abstract

Hydrogels (gels) are attractive tools for tissue engineering and regenerative medicine due to their potential for drug delivery and ECM-like composition. In this study, we use rheology to characterize GelMA/alginate gels loaded with human platelet lysate (PL). We then characterize these gels from a physicochemical perspective and evaluate their ability to transport PL proteins, their pore size, and their rate of degradation. Finally, their biocompatibility is evaluated. We describe how alginate changes the mechanical behavior of the gels from elastic to viscoelastic after ionic (calcium-mediated) crosslinking. In addition, we report the release of ~90% of PL proteins from the gels and relate it to the degradation profile of the gels. Finally, we evaluated the biocompatibility of the gels. Thus, the developed gels represent attractive substrates for both cell studies and as bioactive materials.

## 1. Introduction

The extracellular matrix (ECM) is a three-dimensional network of proteins and glycoproteins surrounded by cells. The ECM provides the physical and biochemical support that allows cells to carry out important processes such as proliferation and intracellular communication. An important feature of the ECM is its viscoelasticity, as it responds to stress and deformation in a time-dependent manner. Therefore, novel biomaterials that mimic the biochemical complexity of the ECM and also allow for tunable mechanical features are needed to support cellular functions and provide adequate physical support [1]. In recent years, hydrogels (gels)—hydrophilic networks of polymers—have been widely studied for their broad potential in regenerative medicine due to their exploitation as drug-delivery tools that are also capable of resembling the ECM thanks to their biological tissue resemblance [2,3]. Many polymers of natural origin have been used to make gels. Gelatin, for example, is a polypeptide obtained from the hydrolyzation of collagen, that has been widely used in tissue engineering due to its biocompatibility, lack of immunogenicity, and good biodegradability [4,5]. It can form gels due to its temperature-dependent helix-to-coil transition. When the system temperature is lowered, the polymer reverts to the triple helix structure of collagen mediated by hydrogen bonds. However, if the temperature is increased, the hydrogen bonds are broken, and there is a loss of organization in the hydrogel structure, characterized by a transition from gel to solution [6]. To circumvent this limitation, chemical crosslinkers such as glutaraldehyde have been used, but these are associated with increased cytotoxicity [7,8]. Gelatin methacryloyl (GelMA) prepared via gelatin methacrylation has the advantage of being photocrosslinkable after the addition of a photoinitiator (e.g., Irgacure 2959, LAP, etc.) [9,10]. Photo-crosslinked GelMA gels are non-toxic and still carry the arginine–glycine–aspartatic acid (RGD) motifs required for cell adhesion. GelMA was first developed and described by Van Den Bulcke et al. and has since been widely used in tissue engineering [11,12,13,14]. Alginate or alginic acid is a polysaccharide extracted from brown algae and is also widely used in regenerative medicine and tissue engineering. It exhibits good biocompatibility properties and is usually ionically cross-linked by divalent cations such as Ca^2+^, resulting in the characteristic “eggbox” structure [15]. Some disadvantages prevent alginate from being used by itself. First, shaping the hydrogel is difficult because immersing the alginate solution in a calcium bath causes immediate cross-linking of the alginate chains, and second, there is a lack of cell adhesion sites. In addition, the commonly used phosphate buffers (e.g., PBS) lead to rapid degradation of alginate gels due to the formation of calcium phosphates, resulting in the loss of hydrogel structure [16,17]. To circumvent these limitations, alginate is often blended with other polymers. In fact, GelMA/alginate blends have been used for 3D printing and tissue engineering. The addition of alginate to GelMA gels improves the mechanical strength of the gel by crosslinking an additional polymer while simultaneously embedding the alginate into the desired shape and introducing cell anchoring points. Most importantly, it has been shown that by adding alginate to GelMA gels, it is possible to control the viscoelasticity of the gel—in contrast to the almost purely elastic behavior of GelMA [18]. Apart from providing a platform for cell adhesion, GelMA and alginate blends are not bioactive by themselves since they do not possess any biological effect. Indeed, many commonly used polymers do not provide reliable cell adhesion and require further functionalization to achieve it (e.g., fibronectin coating) to be used either as biomaterials or as substrates for cell studies [19,20]. Therefore, the resulting matrices rely on the introduction of other bioactive components, such as proteins, to induce a biological response [21,22,23].

To introduce bioactivity, we incorporated human platelet lysate (PL) into the gel. PL is a promising hemoderivative derived from platelets and is rich in chemokines and growth factors (GFs), including epidermal growth factor (EGF), transforming growth factor β (TGF-β), and vascular endothelial growth factor (VEGF) [24]. PL components are involved in cell proliferation, cell differentiation, angiogenesis, and wound healing, making PL a very attractive medium for regenerative medicine purposes. Indeed, gels have already been used as a method for the delivery of PL-derived GF and chemokines [25,26]. GelMA gels have also been reported to exhibit improved mechanical properties after the addition of PL, while inducing differentiation of mesenchymal stem cells toward bone [27]. In the present work, we describe for the first-time double-network gels formed of GelMA and alginate functionalized with PL (GAPL) as drug-delivery systems. This double-network approach, which consists of the formation of two independently crosslinked polymer networks, allows us to first prepare a GelMA network in which the alginate chains are entrapped and then crosslinked with CaCl_2_ within the GelMA network [28,29]. The effects of alginate concentration on the mechanical behavior of the gel, as well as its biocompatibility, were also investigated.

## 2. Materials and Methods

Materials. Alginic acid sodium salt (CAS 9005-38-3) and penicillin–streptomycin were purchased from Sigma-Aldrich Chemical Co. (Saint-Quentin-Fallavier, France). Trypsin (Gibco), Dulbecco’s Modified Eagle Medium (HyClone™ DMEM), Fetal Bovine Serum (HyClone™), AlamarBlue kit, LDH cytotoxicity assay (Invitrogen), DPBS (Gibco), and BCA test were purchased from Fisher Scientific (Illkirch, France). GelMA lyophilizate (degree of substitution 50%) and lithium phenyl-2,4,6-trimethylbenzoylphosphinate (LAP) were purchased from BICO (Cellink, Gothenburg, Sweden). Human platelet lysate was purchased from Macopharma (Tourcoing, France). Calcium chloride anhydrous was purchased from Carlo Erba (Milano, Italy).

Hydrogel solution preparation. Solutions of GelMA, alginate (Alg), and LAP were prepared as follows. GelMA (275 mg) and Alg (0–150 mg) were dissolved in 2.5 mL of human platelet lysate (PL) and 2.5 mL of deionized water in a beaker heated to 40 °C. After complete dissolution of the polymers, LAP photoinitiator (5 mg) was added in the dark. Gels from these solutions were then prepared differently for each experiment. The final concentrations of each component were GelMA 5.5% *w*/*v*, Alg 0–3% *w*/*v*, LAP 0.1% *w*/*v*, human platelet lysate 50% *v*/*v*, and deionized water 50% *v*/*v*. Gels were designated GAxPL, where x indicates alginate concentration (i.e., GA2PL for gels made with alginate 2% *w*/*v*). Gels without alginate (0% *w*/*v*) are referred to as GelMA- PL gels. Similarly, gels prepared without platelet lysate (deionized water 100% *v*/*v*) are referred to as GA2 gels.

Rheological measurements of uncrosslinked and crosslinked gels. Rheological measurements were performed by means of an HAAKE MARS III rheometer (Thermo Fisher Scientific, Dreieich, Germany). To study the kinetics of the photocrosslinking of the uncrosslinked hydrogel solutions, the tests were carried out in oscillatory conditions with a custom setup. A UV module consisting of the main body with integrated mirrors and a quartz glass plate (Ø = 20 mm) (Thermo Fisher Scientific, Dreieich, Germany) was used to study the kinetic of photocrosslinking. To irradiate the sample through the mirrors, a mount with a UV lamp (405 nm, 40 W) was installed. The distance between the light source and the sample was measured to be ~8 cm. Time sweep measurements were performed by recording the storage, G′ (which measures the elastic component of the material), and loss, G″ (which measures the plastic component of the material), moduli at constant deformation, γ, of 1% and frequency, ν, of 1 Hz at *t* = 600 s and T = 25 °C. To determine the mechanical properties of the crosslinked gels, mechanical tests have been performed in oscillatory conditions. Hydrogel solutions were prepared as previously described and subsequently poured into a glass Petri dish (Ø = 30 mm). After, the gels were UV-crosslinked for 3 min. Gels were then cut by means of an Ø = 20 mm punch (Gedore, Remschied, Germany). UV-only gels were tested directly after the punch. To study the effect of CaCl_2_ crosslinking, UV-crosslinked gels were then submerged in a CaCl_2_ 0.09 M bath for 15 min (UV + Ca GAxPL gels). UV + Ca gels were carefully dried with a paper towel prior to analyses. The gap between plates was adjusted by means of short stress sweep tests (ν = 1 Hz; stress range 1–5 Pa) until a consistent G′ at all datapoints was reached. After gap optimization, frequency sweep tests (τ = 1 Pa; ν = 0.01–100 Hz) and stress sweep (ν = 1 Hz; 1 Pa < τ < 10,000 Pa) were then performed at T = 37 °C. Tests were performed in a humidified environment to avoid water evaporation using a sample hood (ThemoFisher, Karlsruhe, Germany) and placing samples inside with a wet paper cloth. All samples were analyzed once and then discarded.

Absorbing capacity and degradation. To evaluate degradation and absorbing capacity of the gels, UV-only and UV + Ca-crosslinked GA2PL gels were prepared as described previously. Gels were cut by means of an 8 mm diameter biopsy punch (Gima, Longiano, Italy). Gels’ initial weights were recorded, and then gels were transferred to a 24-well plate and incubated in 2 mL of DPBS at 37 °C. Weight measurements were taken at 1, 2, 7, 14, 21, and 28 days. Absorption/degradation of the gels was determined via the following formula:

$$∆Weight\;\left(\%\right)=\frac{\left(W_{t}*100\right)}{W_{0}}$$

where *W_t_* is the weight recorded at a given timepoint and *W*_0_ is the initial weight.

Protein release quantification. Bicinchoninic acid (BCA) test was performed to evaluate protein release from the gels. GA2PL gels were prepared as previously described and cut by means of a 6 mm diameter biopsy punch (Gima, Longiano, Italy), whereas GA2 gels (deionized water 100% *v*/*v*) were used as controls. The average gel volume was calculated to be ≈0.113 mL, resulting in a total volume of human platelet lysate ≈0.056 mL per gel. The gels were then incubated in 2 mL of DPBS. After 1, 2, 3, 4, 6, 24, 96, 120, 144, and 192 h, 1 mL of the solution was collected and replaced with fresh DPBS. BCA test was performed accordingly to manufacturer’s protocol on both GA2PL and GA2 gels, as well as on 1:100 dilutions of human platelet lysate. Human platelet lysate protein concentration was found to be 54.29 mg mL^−1^. Calculated protein release from GA2 gels was subtracted from GA2PL-calculated protein release.

Scanning electron microscopy (SEM). The morphology of UV + Ca crosslinked GA2PL gels was observed by means of an SEM microscope (Quanta 250 FEG FEI, Thermo Scientific, USA). UV + Ca-crosslinked GA2PL gels were prepared as described previously and cut by means of a 20 mm punch (Gedore, Dreieich, Germany). Samples were first fixed in glutaraldehyde (2% *w*/*v*) for 24 h and then dehydrated in an increasing ethanol gradient (30, 80, 100% *v*/*v*) for 2 h in each solution and finally dried at a critical point [30]. All samples were coated with 6 nm of platinum prior to analyses. All observations were made in the secondary electron emission mode with a high voltage of 5 kV.

Cell culture. Vero green monkey kidney cells (ATCC CCL-81) were cultured in DMEM High Glucose supplemented with 10% *v*/*v* bovine calf serum and 1% *v*/*v* penicillin–streptomycin. Media were discarded and changed every 2 days. Cells were incubated at T = 37 °C in a humidified atmosphere of 5% CO_2_.

Evaluation of gels biocompatibility. Sterile GA2PL gels for in vitro tests were prepared as follows: GelMA, Alg, and LAP photoinitiator were dissolved as previously described, and then the warm solution was loaded into a syringe and sterilized via filtration (cellulose acetate, pore size 0.2 μm) (Sartorius AG, Goettingen, Germany). Then, the mixture was poured into a Petri dish (Ø = 30 mm) and UV-crosslinked for 3 min. Subsequently, the gels were cut by means of an 8 mm diameter biopsy punch (Gima, Longiano, Italy). Ionic crosslinking of alginate was finally achieved by submerging the resulting cylinders into a sterile calcium chloride 0.09 M solution for 15 min. The height of each cylinder was measured to be ≈3.5 mm. After the crosslinking, gel cylinders were rinsed with sterile deionized water to remove excess CaCl_2_. Cells were plated in a 24-well plate (cell density: 13,500 cells/well) and, after 24 h, the medium was discarded, and the cells were quickly rinsed with DPBS. Next, 2.0 mL of fresh medium was added in each well, and gels were placed on top of the cell layer (V_medium_/V_gel_ = 19). As a positive control for cell cytotoxicity, medium supplemented with Triton X-100 (0.1% *v*/*v*) was employed, while untreated cells were regarded as negative control. After 24 h of incubation, AlamarBlue and LDH tests were performed according to manufacturer protocols. Gels’ biological evaluation was performed according to ISO 10993-5 guidelines [31].

Statistical analysis. Biological results were analyzed via one-way ANOVA (analysis of variance), followed by Tukey’s HSD post hoc range test, to assess differences between the different groups in GraphPad Prism 8. Significant differences were considered at *p* < 0.05.

## 3. Results and Discussion

**Figure 1:**
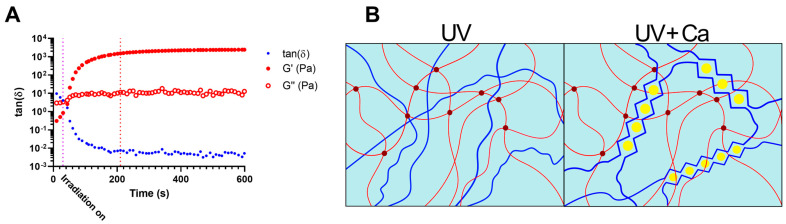
Crosslinking mechanism of the gels. (**A**) Dependence of the loss tangent, tanδ ($G\prime \prime /G^{\prime }$), on time GA2PL solutions are exposed to UV light (λ = 405 nm). UV light was turned on 30 s after the beginning of the measurements (purple dotted line, t = 30 s). After ~180 s, crosslinking reached completion (red dotted line, t = 210 s). (**B**) Sketched representation of gels’ double networks after UV and UV + Ca crosslinking. Red lines: GelMA chains; dark red dots: UV-mediated covalent crosslinks; blue lines: alginate chains; yellow dots: calcium ions. Experimental conditions: GelMA, 5.5% *w*/*v*; alginate, 2% *w*/*v*; LAP, 0.1% *w*/*v*; human platelet lysate, 50% *v*/*v*. Oscillatory time sweep experiments were performed at T = 25 °C.

GelMA 5.5% *w*/*v*, alginate 2% *w*/*v*, and human platelet lysate (GA2PL) solutions were prepared to study the UV-mediated gelation kinetics. Indeed, finding the optimal crosslinking time is crucial, as over exposure to light might have adverse effects on polymers, proteins, and cells [32]. In an effort to optimize UV crosslinking time, UV kinetics were performed on GA2PL solutions. The storage moduli (G′) and loss moduli (G″) were recorded as a function of time. Thirty seconds after the start of the analysis, the UV lamp (λ = 405 nm) was turned on. After ~180 s of UV irradiation, the storage modulus reached a plateau, indicating the successful formation of a covalently bound GelMA network (Figure 1A). The temporal decrease in the loss tangent tanδ($G^{\prime \prime }/G^{\prime }$) indicates the progressive formation of a network driven by elastic forces: the storage modulus (G′) increased considerably, while the loss modulus (G″) increased only slightly. The crosslinking time is consistent with the expectations arising from the degree of substitution of the GelMA used (50%) and the use of LAP as a photoinitiator [33,34,35]. To further crosslink the UV-crosslinked gels, the gels are immersed in a 0.9 M CaCl_2_ solution for 15 min, which induces ionic crosslinking of the alginate chains (Figure 1B).

**Figure 2:**
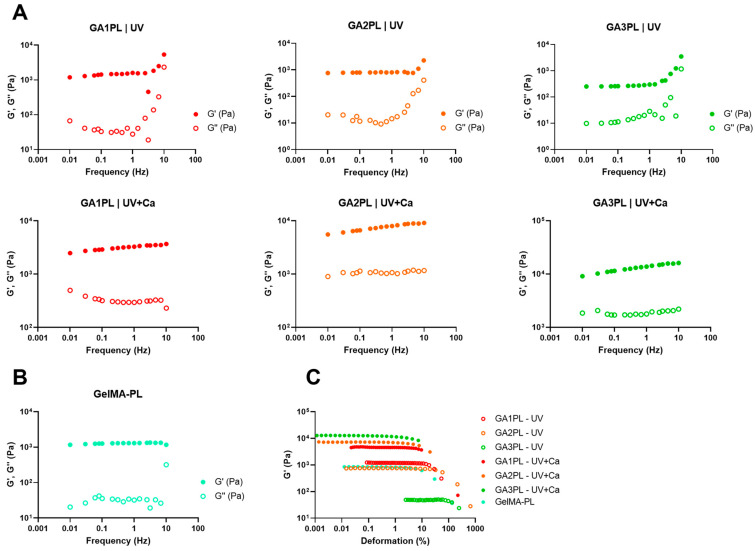
Rheological analysis of GAxPL/GelMA-PL gels at different alginate concentrations (0–3% *w*/*v*). (**A**,**B**) Mechanical spectra obtained from frequency sweep analyses of gels represented as the elastic (G′) and viscous (G″) moduli over frequency (Hz) (colored full dots: G′; colored empty dots: G″). (**C**) Elastic modulus (G′) over deformation of gels before (UV-only) and after calcium crosslink (UV + Ca) (colored empty dots: UV-only gels; colored full dots: UV + Ca gels). Experimental conditions: GelMA, 5.5% *w*/*v*; alginate, 1–3% *w*/*v*; LAP, 0.1% *w*/*v*; human platelet lysate, 50% *v*/*v*. Frequency and stress sweep experiments were performed at T = 37 °C.

We then investigated the effects of alginate concentration on the overall mechanical response of the gels before and after calcium crosslinking. For this purpose, we changed the alginate concentration in the range of 0–3% *w*/*v*. Frequency sweep tests revealed elastic gels for GelMA-PL and UV-only crosslinked gels, as shown by the independence of elastic modulus from frequency at low frequencies (<1 Hz) (Figure 2A,B). On the other hand, UV + Ca-crosslinked gels exhibited viscoelastic properties, as shown by the increasing dependence of Gꞌ values on frequency, and this is also confirmed by the strain-softening behavior at high deformations (Figure 2A,C). Interestingly, the storage modulus of the UV-crosslinked GAPL gels exhibited nonlinear behavior at frequencies > 4.64 Hz, as evidenced by an increase in both elastic and storage moduli. This increase was present in gels containing non-crosslinked alginate but was absent in GelMA-PL gels (Figure 2B). This phenomenon is similar to that observed in gels prepared with highly acetylated chitosan of different molecular weights and crosslinked with genipin [36]. Interestingly, it is also present in Matrigel, an ECM protein-based matrix commonly employed to produce organoids [37,38,39]. However, this behavior is not found in UV + Ca crosslinked gels, hence suggesting it being a result of physical entanglements of free semi-rigid alginate chains. However, the matter requires further study also comparing platelet-lysate-free hydrogels.

The mechanical spectra obtained from the frequency sweep experiments were analyzed in terms of the Maxwell model. The G′ and G″ moduli obtained from the measurements were fitted as a function of angular frequency (ω) using the following equations (Equations (1) and (2)), which represent a series of springs composed by a sequence of springs and dashpots connected in parallel and a purely elastic spring (G_e_) [40]:

(1) G′=Ge+∑i=1nGi(λiω)21+(λiω)2; Gi=ηiλi

(2) G″=∑i=1nGiλiω1+(λiω)2; Gi=ηiλi

where n is the number of Maxwell elements, $G_{i}$ is the spring constant, and $\eta_{i}$ and $\lambda_{i}$ are, respectively, the dashpot viscosity and the relaxation time of the i-th Maxwell element. To determine the correct number of Maxwell elements to take into consideration, a statistical procedure to reduce $X^{2}\cdot N_{p}$, where $X^{2}$ is the sum of squared errors and $N_{p}(2+n)$ is the number of Maxwell elements, was performed. Relaxation times were considered dependent from each other and arbitrarily scaled by a factor of 10 [41]. We exploit this model to determine the shear modulus, G, of the gels via the following Equation (3):

(3) $$G=G_{e}+\sum_{i=1}^{n}G_{i}$$

**Figure 3:**
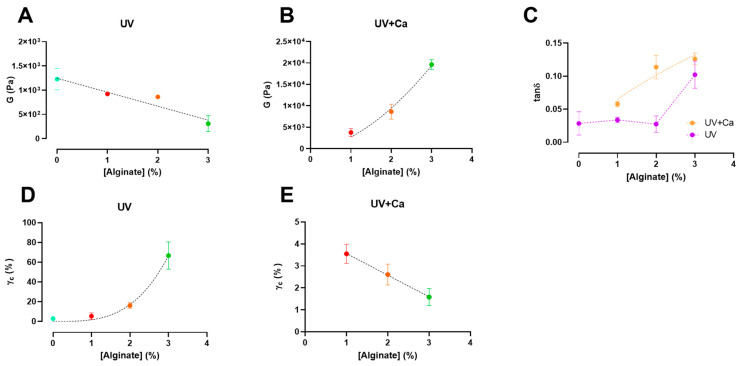
Alginate concentration has different effects on shear modulus (G) and critical strain (γc) pre- and post-ionic crosslinking. (**A**,**B**) Shear modulus (G) of UV-crosslinked and UV + Ca crosslinked gels as function of alginate concentration. (**C**) Dependence of the loss tangent (tanδ) at ν = 1.0 Hz of covalent (UV) and covalent + ionic (UV + Ca) gels obtained from frequency sweep tests over different alginate concentrations. (**D**,**E**) Critical strain, γ_c_, of UV-crosslinked and UV + Ca crosslinked gels as function of alginate concentration. Experimental conditions: GelMA, 5.5% *w*/*v*; alginate, 0–3% *w*/*v*; LAP, 0.1% *w*/*v*; human platelet lysate, 50% *v*/*v*. All frequency sweep and stress sweep measurements were performed at T = 37 °C, *n* = 3. Data are reported as mean ± s.d.

The shear modulus calculated this way reflects the shear modulus of gels under constant stress at small deformations (Figure 3A,B). The elastic modulus, G′, and the deformation, γ, values obtained from stress sweep data were successfully fitted by means of the Soskey–Winter Equation (4) [42]:

(4) $$G^{\prime }={G^{\prime }}_{0}\frac{1}{1+{\left(b\gamma \right)}^{n}}$$

where ${G^{\prime }}_{0}$ is the limiting value of the storage modulus for γ→0, whereas b and n are variable parameters. The critical strain, defined as the strain that triggers the strain softening, was determined as γC=G′G′0=0.95 [43].

We report that the calculated shear moduli (G) (Equation (3)) of UV-crosslinked gels are inversely proportional to the alginate content (Figure 3A). Indeed, pure GelMA-PL networks exhibit the highest shear modulus, which decreases almost linearly with increasing uncrosslinked alginate concentration. This downward trend could be attributed to the increasing amount of alginate chains, which could affect the formation of a fully crosslinked GelMA network. However, when the gels are ionically crosslinked via CaCl_2_, the shear moduli in GA1PL, GA2PL, and GA3PL gels increase 2.6-, 8.7-, and 63.8-fold, respectively, compared to their purely covalent counterparts (i.e., UV gels). Indeed, the calculated shear moduli of UV + Ca gels were found to scale accordingly to G ∝ [alginate]^1.65^ (Figure 3B). On the other hand, alginate concentration increased the critical strain values of the photo-crosslinked-only gels determined via Soskey–Winter fitting. The critical strain values of these gels scaled according to γ_c_ ∝ [alginate]^3.35^, indicating that higher alginate concentrations delayed the onset of strain softening (Figure 3D). Again, this behavior could be attributed to the progressive formation of a partially incomplete GelMA network as the methacrylated lysines are masked by increasing amounts of alginate. After crosslinking alginate with calcium, the critical strains for UV + Ca-crosslinked gels were found to be inversely proportional to the alginate content and decreased linearly, as higher alginate concentrations lead to an early rupture of the network (Figure 2B and Figure 3E).

We then calculated the loss tangent (tanδ = G″G′) at 1.0 Hz from the frequency sweep data. The results presented in Figure 3C show an increase in viscoelasticity of the hydrogel as a function of alginate concentration. For UV-crosslinked networks, the increase in viscoelasticity follows the threshold of [alginate] ≥ 3% *w*/*v*, while for UV + Ca gels a proportional increase equal to tanδ ∝ [alginate]^0.45^ is observed. These results demonstrate the possibility of adjusting the viscoelasticity of the gels by changing the alginate concentration. It is evident that the nature of the gel before ionic crosslinking is predominantly elastic—as expected from a covalently bonded polymer network—but after ionic crosslinking, the nature of the network shifts toward higher viscoelasticity, in agreement with the reports of Chen et al. [14]. This behavior makes our hydrogels attractive substrates, as they have both cell adhesion motifs and adjustable viscoelasticity [27,28].

Gels were functionalized with human platelet lysate (PL) because of their promising biological potential for biomaterials. To evaluate protein release, we performed a BCA assay with the UV + Ca GA2PL gels. First, we determined the protein content of the fresh PL—which was 54.3 mg mL^−1^. Protein release occurs in three phases: (I) a first phase (between 0 and 6 h) in which ~30% of the protein content is released; (II) a second phase (between 6 and 24 h) in which only another ~7% of the proteins are released; and finally (III) a third phase (from 24 to 192 h) in which the release rate increases again and another ~56% of the proteins are released.

These three phases are attributed to the different mechanisms. The first phase of release may correspond to proteins such as growth factor released via diffusion through the porous network of gels, while the third phase of protein release is associated with partial degradation of the network. The second phase is the result of the overlap of both mechanisms. A similar relationship between swelling/degradation and release of proteins has been described for dexstran-based hydrogels and PEG [44,45]. PEG hydrogels subjected to reverse gelation caused a second increase in release after an initial plateau, similar to what we reported.

**Figure 4:**
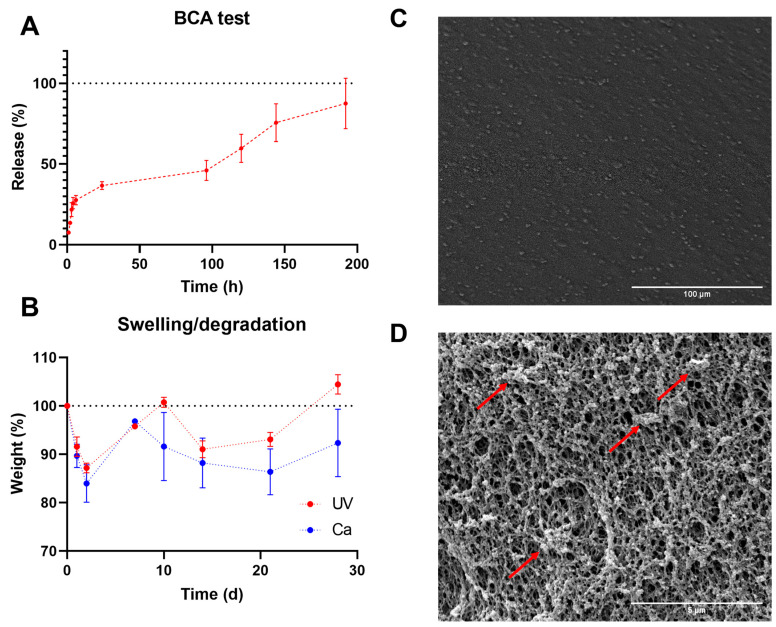
GA2PL gels’ characterization. (**A**) BCA test to quantify protein released from UV + Ca-crosslinked GA2PL over 8 days. Red dotted line drawn to guide the eye. (**B**) Swelling and degradation test of UV- and UV + Ca-crosslinked gels. Blue and red dotted lines drawn to guide the eye. (**C**,**D**) SEM images of the surface of GA2PL gels (magnification: 500× and 10,000×, respectively). Red arrows: globular structures attached to the polymer chains. Experimental conditions: GelMA, 5.5% *w*/*v*; alginate, 2% *w*/*v*; LAP, 0.1% *w*/*v*; human platelet lysate, 50% *v*/*v*. Data are shown as mean ± s.d., *n* = 4 replicates.

This mechanism can indeed be associated with the absorption/degradation experiments. As can be seen in Figure 4B, both UV- and UV + Ca-crosslinked GA2PL gels first undergo a degradation process (days 1 and 2). Since both the pure UV and UV + Ca gels follow the same pattern in the first days of degradation, the process cannot be attributed to the formation of calcium phosphate and the resulting release of alginate, even though the gels were immersed in DPBS. The phosphate present in DPBS would bind calcium ions to the alginate and cause partial degradation—which could explain the different final values (day 28). Overall, little degradation is observed for UV + Ca gels. SEM images show a uniform morphology of the gel surface (Figure 4C). We then examined the cross-section of the gels, which showed a fully formed network with an average pore size of 0.230 ± 0.03 μm. In particular, spherical structures adhering to the polymer chains were evident, which were identified as proteins from platelet lysate (Figure 4D).

**Figure 5:**
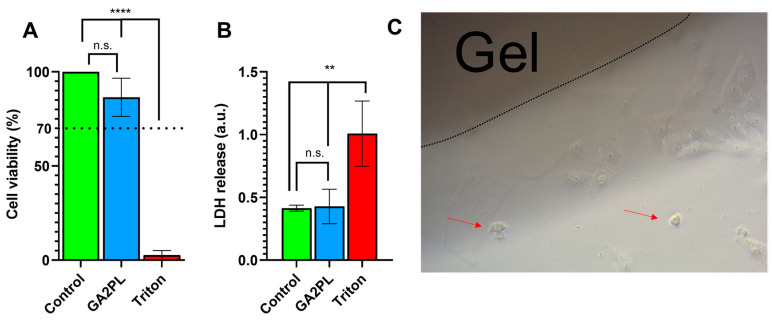
Cytotoxicity evaluation of UV + Ca-crosslinked GA2PL gels. (**A**,**B**) AlamarBlue and LDH tests to check effects cytotoxicity of gels placed atop the cell layer. Black dotted line: 70% threshold for cytotoxicity. (*n* = 3–5 replicates for each experimental condition; statistics: One-way ANOVA followed by Tukey’s HSD post hoc range test between all groups was employed (****, *p* < 0.0001; **, *p* < 0.05; n.s., not significant). (**C**) Optical microscopy of Vero cells in the presence of UV + Ca-crosslinked GelMA/alginate gels. Red arrows indicate few suffering cells (≪20%).

Finally, to evaluate the potential of the gels as biomaterials, we performed AlamarBlue and LDH assays on green monkey kidney Vero cells to assess the effects of the gels on cell metabolism and membrane damage. Briefly, the gels were placed on the cell layer, and after 24 h, the metabolic activity of the cells and membrane lysis were assessed. No cytotoxicity was detected, as the viability of the cells was ≥85% compared with the control group (untreated cells) (Figure 5A). In addition, no statistical difference in LDH release was detected between cells with and without gel (Figure 5B). Qualitative (optical microscopy) analysis of the cells revealed very few (≪20%) suffering cells, indicating that the gels had no cytotoxicity according to the standard ISO 10993-5 (score: 0; reactivity: none) (Figure 5C) [19].

## 4. Conclusions

In this work, we prepared GelMA/alginate gels functionalized with platelet lysate (PL) because it has attractive properties for tissue engineering. We investigated the gels mechanical behavior. We found that gels cross-linked with light only (e.g., UV light only) predominantly exhibited elastic behavior characteristic of covalent hydrogels. However, after ionic crosslinking of alginate by calcium, such gels acquire viscoelastic properties. This was confirmed by the time-dependent response of the gels to stress/strain and by their strain-softening behavior. We have shown that alginate can be used not only to tune mechanical performance (e.g., shear modulus and critical strain), but that it also contributes crucially to viscoelasticity in otherwise almost completely elastic networks. In addition, the mechanical spectra of pure UV hydrogels reveal the possibility of the formation of entanglements between the alginate chains under constant loading at different frequencies, but this hypothesis requires further study and is outside the scope of this article. We have also shown that the prepared gels loaded with platelet lysate are capable of releasing proteins (e.g., growth factors, chemokines)—which are of interest because of their wound healing and differentiation potential and because their release depends on both the porosity of the network and its degradation rate. Gels prepared in this way have small pores and can be degraded over time in physiological medium (PBS). Finally, the gels exhibited satisfactory biocompatibility, making them suitable for biomaterials and tissue-engineering purposes.

## Data Availability

Data from this article are available upon reasonable request.
